# Supplementary material for: Competing covalent and ionic bonding in Ge-Sb-Te phase change materials
Source: Sci Rep. 2016 May 19;6:25981. doi: 10.1038/srep25981 (PMC4872153; doi:10.1038/srep25981)
Supplement: Supplementary Information [file srep25981-s1.pdf]

# Supplementary Information

## For

## Competing ionic and covalent bonding in Ge-Sb-Te phase change materials

Saikat Mukhopadhyay[1*], Jifeng Sun[1], Alaska Subedi[2], Theo Siegrist[3], David J. Singh[4]

[1]Materials Science and Technology Division, Oak Ridge National Laboratory, Oak Ridge, TN 37831-6056 USA

[2]Max Planck Institute for the Structure and Dynamics of Matter, Hamburg, Germany

[3]Department of Chemical and Biomedical Engineering, FAMU-FSU College of Engineering, Tallahassee, FL 32310, USA

[4]Department of Physics and Astronomy, University of Missouri, Columbia, MO 65211-7010 USA

*Corresponding author: (email) mukhopadhyas@ornl.gov

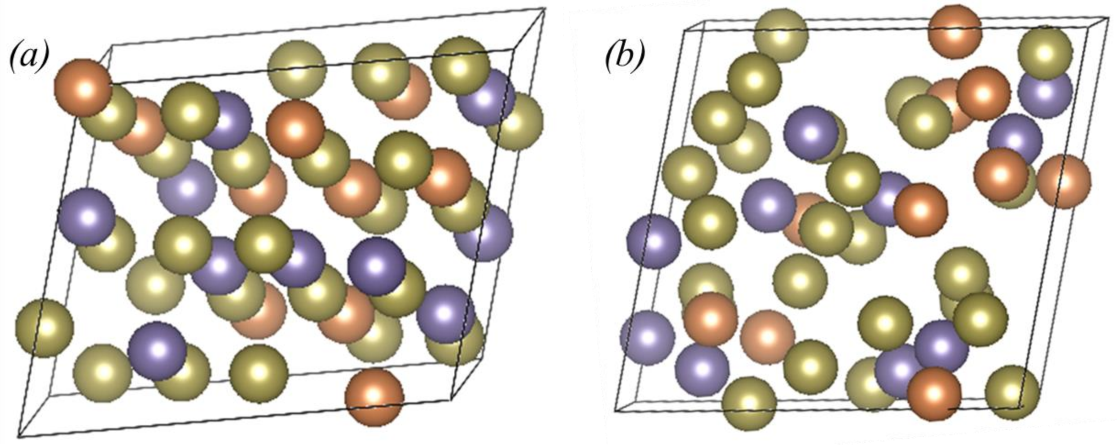


Figure S1: Optimized configuration of (a) c-GST and (b) a-GST. The violet, orange and brown spheres represent the Ge, Sb and Te atoms.

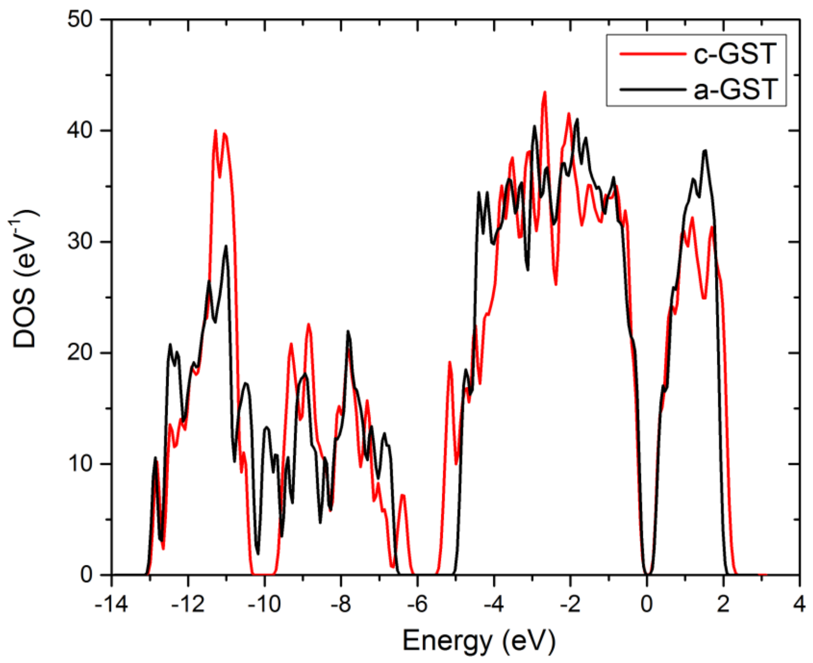


Figure S2: Total density of states of c-GST (red line) and a-GST (black line) calculated with PBE-GGA.

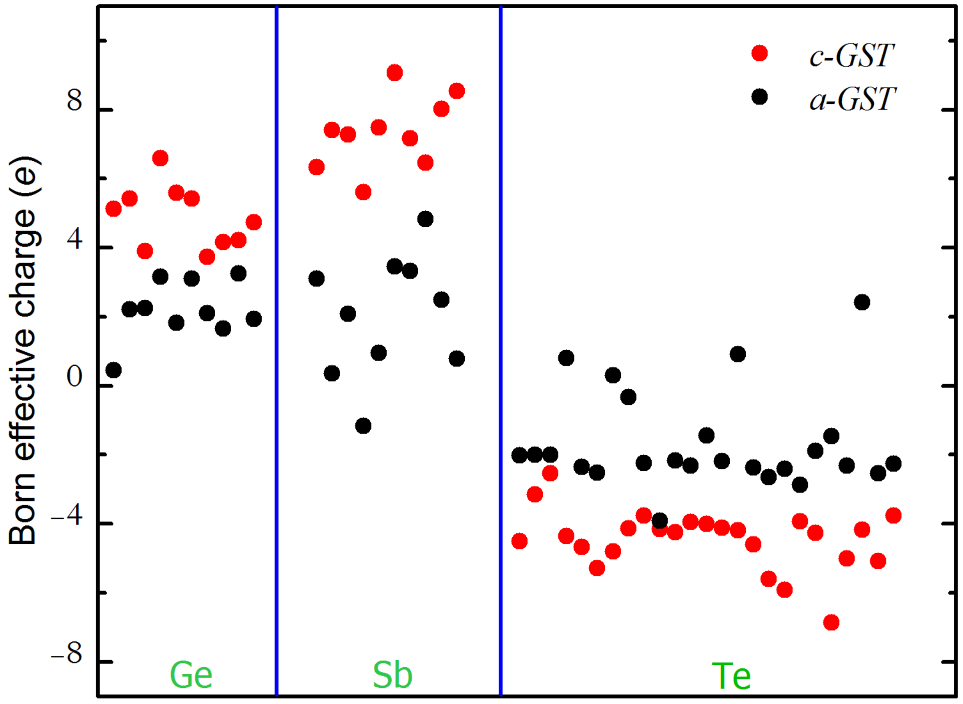


Figure S3: Born effective charges on Ge, Sb and Te atoms in c-GST and a-GST. Red and black spheres stand for Born effective charges in c-GST and a-GST, respectively.

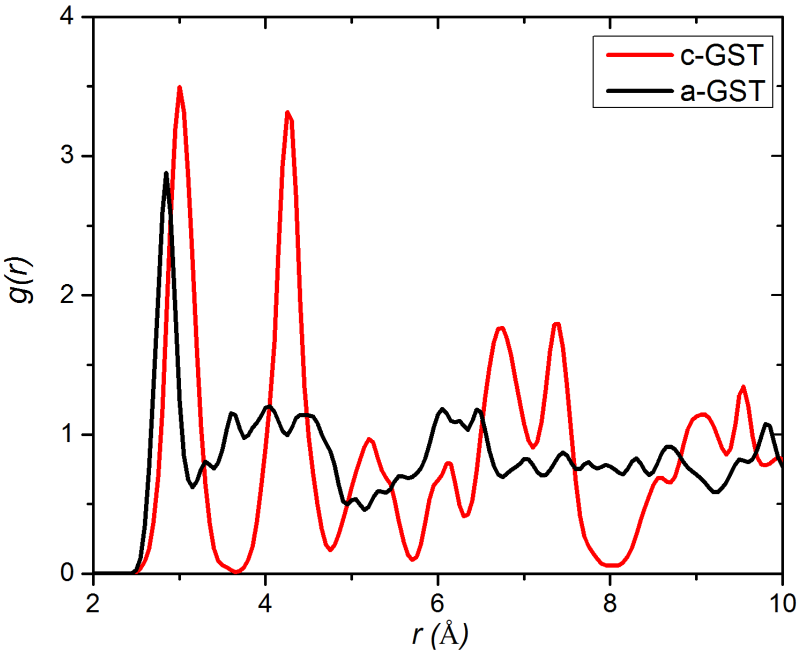


Figure S4: Pair correlation functions for c-GST and a-GST.
